# Prospects for Physical Therapy in Rheumatoid Arthritis: Literature Review

**DOI:** 10.3390/healthcare14172751

**Published:** 2026-08-28

**Authors:** Dana Taizhanova, Olga Pokrashenko, Anna Shalygina

**Affiliations:** Department of Internal Medicine, Karaganda Medical University NC JSC, Karaganda 100008, Kazakhstan; tayzhanova@qmu.kz (D.T.); dr.shalygina1985@gmail.com (A.S.)

**Keywords:** rheumatoid arthritis, cryotherapy, magnetotherapy, laser therapy, ultrasound therapy, neuromodulation

## Abstract

**Background**: The incidence of rheumatoid arthritis is steadily increasing worldwide, resulting in a growing number of individuals who may lose the ability to perform self-care activities and professional duties, thereby reducing their quality of life. Rheumatoid arthritis represents a significant social problem, limiting the ability of the working population to lead a full life. A comprehensive approach to the treatment of patients with rheumatoid arthritis, including both pharmacological therapy and an individualized rehabilitation program, is of strategic importance for maintaining their functional status. **Materials and Methods**: A review of modern physiotherapy methods (physical factors) in patients with rheumatoid arthritis was conducted in the international scientific databases Web of Science, PubMed, Google Scholar, and Elibrary over a seven-year period to identify an alternative approach to the treatment of this disease. **Results**: The search identified 12 articles presenting the results of clinical studies (3), cross-sectional study (1) and randomized clinical trials (8). These studies investigated physiotherapy interventions in patients with RA and highlighted the potential value of exploring alternative treatment approaches for this patient population. Despite substantial heterogeneity in the interventions, treatment protocols, and combinations of physical factors used, all of the reviewed methods demonstrated positive effects. **Conclusions**: Physiotherapy interventions may have a positive impact on the clinical course of rheumatoid arthritis; however, the available evidence is insufficient to draw definitive conclusions regarding their effectiveness, primarily due to the lack of long-term follow-up data and substantial heterogeneity in the number and design of treatment sessions, as well as in the physical factors applied. The long-term effects of physiotherapy interventions remain insufficiently studied and warrant further research aimed at developing standardized and evidence-based treatment protocols.

## 1. Introduction

Rheumatoid arthritis is an autoimmune progressive disease that involves both physical and psychological factors. These factors reflect possible changes in emotional well-being (restlessness, anxiety, and depression), which may exacerbate the course of the underlying condition, namely, changes in pain sensitivity, increased duration of morning stiffness, and impaired adherence to treatment [1]. Currently, the etiology of rheumatoid arthritis remains not fully understood. Preclinical factors that may trigger the development of the disease include working in the agricultural and industrial sectors, antibiotic therapy, obesity, smoking, and genetic predisposition [2]. Thus, the search for etiological factors influencing the manifestation of this disease remains a priority. The clinical presentation varies. It is characterized by severe symmetrical pain and damage to the osteoarticular apparatus, irreversible joint deformities, extra-articular complications, and neurological symptoms, leading to loss of working capacity and self-care, a decrease in quality of life, and psychological instability [3,4]. Drug treatment plays a key role in the treatment of rheumatoid arthritis.

Comorbid conditions in patients with rheumatoid arthritis must also be taken into account [5,6]. There are also studies that suggest a role for pro-inflammatory cytokines in the development of depression and cognitive impairment [7,8]. Consequently, maintaining minimal disease activity or remission is of the utmost importance: the functioning of other organs and systems directly depends on the progression of RA.

In fact, modern laboratory diagnostic methods are constantly being improved. The study by Chao-Yi Wu et al. focused on antibodies to carbamylated proteins, the detection of which may be a marker of high disease activity and may be of interest for early diagnosis [9]. A recent study assessed blood test parameters, such as red blood cell distribution width, which may also indicate disease activity [10]. However, the main parameters for monitoring patients with rheumatoid arthritis remain: complete blood count, assessment of C-reactive protein (CRP), rheumatoid factor (RF), and antibodies to citrullinated peptides (anti-CCP).

The pathogenesis of the disease is characterized by disruption of cellular and humoral immunity, manifested by an imbalance between pro-inflammatory and anti-inflammatory cytokines and impaired regulation of neutrophil and T-regulatory cell activation [11,12]. A number of studies have demonstrated an association between the microbiome of the mucous membranes and development of autoimmune diseases [2,13]. Thus, options for correcting disease activity in patients from a pathogenetic point of view can be considered based on an integrated approach, including drug therapy, a balanced diet, dietary adherence, nutrigenomics, and support for the functioning of organs and systems [14,15,16].

Pain is one of the key symptoms in patients with RA. The mechanisms underlying pain are complex and involve not only the activation of pro-inflammatory cytokines and changes in pain perception, but also a disruption of the normal interaction between the peripheral and central nervous systems [17]. The nature of the pain is also of significant clinical importance, reflecting its duration, location, impairment of normal joint function, and consequently, a reduction in quality of life. This, in turn, leads to an increase in the frequency of non-steroidal anti-inflammatory drug use and the risk of developing drug-induced gastropathy. This fact highlights the need to seek an alternative approach to treating pain and improving patients’ functional capacity. Currently, there are few studies investigating the effects of physiotherapy interventions in patients with rheumatoid arthritis. By “physiotherapy,” we mean the use of physical agents: laser therapy, magnetic therapy, shockwave therapy, ultrasound therapy, cryotherapy/thermotherapy, photomagnetic therapy, and electrotherapy.

For example, there are comprehensive reviews on low-intensity laser therapy, magnetotherapy, and cryotherapy/thermotherapy [18,19,20]. However, the evidence base for these interventions is limited, and the results are inconclusive due to variations in methodology, procedure duration, the number of sessions, and differences in disease activity. These issues remain contentious in clinical practice and raise important questions. This review will examine methods published over the past seven years. It is also important to bear in mind that the impact of coronavirus infection (COVID-19) on the course and manifestation of rheumatoid arthritis remains an unresolved issue. However, some studies suggest that COVID-19 may act as a trigger for the development of autoimmune diseases, including RA [21,22].

### The Aim of This Study Is

(1) A review of the methods of using physiotherapeutic procedures (laser therapy, magnetic therapy, shock wave therapy, ultrasound therapy, thermotherapeutic procedures, photomagnetic therapy and electrotherapy) in patients with rheumatoid arthritis, including randomized controlled trials and clinical trials;
(2) To analyse and evaluate the clinical effects using appropriate scales (VAS, DAS-28 HAQ, etc.)

## 2. Materials and Methods

This study was conducted between July 2025 and May 2026 as a narrative review aimed at identifying clinical trials focusing on physiotherapy interventions (physical modalities, excluding only kinesiotherapeutic interventions) in patients with rheumatoid arthritis. As this was a review study, a comprehensive search strategy was developed to guide the literature search across the selected databases.

This study provides an overview of physical intervention techniques for patients with rheumatoid arthritis published in the international scientific databases Web of Science, PubMed, Google Scholar, and Elibrary over a seven-year period (2019–2026), with the aim of identifying alternative approaches to pain management and improving quality of life.

The following combinations of keywords were used in the search: ‘rheumatoid arthritis and physiotherapy’, ‘rheumatoid arthritis and magnetic therapy’, ‘rheumatoid arthritis and electrotherapy’, ‘rheumatoid arthritis and laser therapy’, ‘rheumatoid arthritis and cryotherapy’, ‘central sensitization and rheumatoid arthritis’, ‘rheumatoid arthritis and ultrasound therapy’, ‘rheumatoid arthritis and magnetic field exposure’, and ‘rheumatoid arthritis and neuromodulation’. These combinations were selected to identify studies examining the relationship between RA and physical intervention methods. The search strategy included the following filters: publication years (2019–2026), open access, clinical trials, randomized clinical trials, and full-text availability. Information was manually selected according to the predefined search strategy. Only studies reporting the results of physical interventions were considered.

### 2.1. Inclusion Criteria

This review considered studies in which the primary outcome was rheumatoid arthritis and the intervention involved physiotherapy. Studies describing a combined intervention (physiotherapy and kinesiotherapy) were also included, as the effect of this intervention was reflected in the outcomes and results and was considered to be of scientific interest. In the PubMed and Web of Science databases, the search query was restricted to the specified keywords and search strategy. In the Elibrary and Google Scholar databases, the search query was restricted to keywords; the search for information included the following parameters: publication title, full-text availability, and journal articles.

### 2.2. Exclusion Criteria

The exclusion criteria were as follows: studies that were not relevant to the topic; duplicate studies; studies that did not match the search keywords; animal studies; studies without open access; studies containing only the keyword “rheumatoid arthritis”; studies available only as abstracts; studies with fewer than 15 participants; and studies related to drug treatment (drug effects).

### 2.3. Data Extraction

Data from the included studies were entered into a spreadsheet, including key parameters: physical factor, treatment parameters, number of sessions, outcomes, and author. A second table was compiled based on specific characteristics, including study type, sample size, international assessment scales, study limitations, and effects.

### 2.4. Analysis of Results

The initial search identified 134 articles in PubMed, 406 in Web of Science, 258 in Elibrary, and 575 in Google Scholar. Each database was carefully analysed for the presence of the specified keywords and relevance to the research objective. Two articles were additionally included following an analysis of the bibliographic references of previously selected studies and were included in this review due to their alignment with the search strategy.

Furthermore, taking into account the search strategy:

- Google Scholar contained general information on RA, other rheumatic diseases and review articles;
- Elibrary also contained general information on rheumatic diseases, review articles, juvenile arthritis and other rheumatic diseases;
- Studies focusing on the pharmacological treatment of RA were excluded from the PubMed and Web of Science databases, other rheumatic diseases, those listing other conditions alongside RA, or those containing only the word ‘RA’ in their titles or abstracts; studies available only as abstracts were also excluded. Subsequently, through a careful selection process, articles were identified whose titles or abstracts contained the specified keywords and the term “RA”. Duplicates were then excluded, as were studies involving animals, articles available only as abstracts, studies not related to physiotherapy in patients with RA, studies describing comorbid conditions in patients with RA, and studies with a small sample size.

## 3. Results

### 3.1. General Information

The search yielded 1375 articles, of which 1306 were excluded because they did not meet the inclusion criteria, resulting in a total of 69 articles. Review of the abstracts of these articles revealed the following: 34 irrelevant articles, 9 duplicates, 9 studies involving animals, and 2 studies due to participant numbers. Additionally, of the 15 accessible articles, 3 studies were available only as abstracts, which also did not meet the inclusion criteria. Therefore, 12 articles were included in the review. The results of the study are presented in Table 1.

### 3.2. Research Characteristics

The search identified 12 articles presenting the results of clinical studies, a cross-sectional study, and randomized clinical trials. A wide range of techniques was identified among the included studies: magnetotherapy, laser therapy, cryotherapy/psammotherapy, ultrasound therapy, electrotherapy, shockwave therapy, and combined treatments. The data from these studies addressed the topic of physiotherapy interventions in patients with RA, highlighting the relevance of exploring alternative treatment methods for this patient group. Given the wide variability among the studies, differences in the methods used to perform the procedures, and their possible combinations, each of the methods mentioned demonstrated positive effects. The included studies comprised 8 randomized clinical trials (RCTs), 3 clinical trials, and 1 cross-sectional study. The maximum number of participants in a single study was 242 [25].

The main outcome measures were reductions in pain and morning stiffness, as well as improvements in quality of life. The highest number of treatment sessions was reported for the neuroimmune modulation of the vagus nerve technique, whereas the lowest number of sessions was reported for cryotherapy [25,26]. Laser therapy was the most frequently used method (n = 4) [24,27,33,34]. Studies using electrical stimulation provided evidence of medium- and long-term effects [23,25]. The results included both standalone and combined approaches, as well as comparisons between procedures within the same study in patients with RA. The combined methods involved a combination of physical agents (magnetotherapy and laser therapy) with kinesiotherapy.

### 3.3. Pain Syndrome

One of the main complaints that adversely affects quality of life and sleep is pain. Research into the mechanism of pain is currently ongoing, as reflected in the search for new links in its pathogenesis and, consequently, the development of insights into the targeted effects of potential therapeutic approaches on the nature of the inflammatory process [17]. The Visual Analogue Scale (VAS) is one of the key diagnostic indicators for assessing this symptom, as evidenced by the studies reviewed [24,26,27,28,29,31,32,34]. This scale allows patients to subjectively assess the severity of their pain and is sufficiently simple to use. The range of treatment methods available to reduce pain intensity includes magnetotherapy, laser therapy, cryo-/thermotherapy and shockwave therapy.

### 3.4. Morning Stiffness

Morning stiffness is an important symptom reported by patients and is commonly assessed in terms of its duration. In 3 studies, including photomagnetotherapy and kinesiotherapy, magnetotherapy and kinesitherapy, and laser therapy, a reduction in the duration of this symptom was observed [27,32,34]. It should also be noted that the number of treatment sessions directly affecting the severity of this symptom ranged from 10 to 24 sessions.

### 3.5. Quality of Life

All the articles analyzed reported positive effects in treating RA symptoms, including reduced pain intensity, shorter morning stiffness, and reduced fatigue. This resulted in improved joint function and quality of life. The methods reviewed differ in their physical parameters (intervention factors); however, each of these alternative approaches aims to improve the patient’s functional status.

### 3.6. Magnetic Therapy

Three of the included studies examined the use of magnetic therapy, one of which was an RCT. However, the methods of administering this procedure differed. For example, in the study by Kostro AM et al., the use of magnetic therapy and laser therapy was compared, with magnetic therapy (alternating sinusoidal bipolar and rectangular bipolar fields) demonstrating an advantage in terms of mobility of the interphalangeal joints [24]. Two of the studies used combined treatment methods. Thus, in the study by Zwolińska J et al., a combined method was used involving kinesiotherapy and magnetic therapy (static magnetic field, flux density 7 mT, and rectangular bipolar pulse with a frequency of 10–20 Hz and a flux density of 7 mT) [32]. Also, one of the combined treatment methods involved kinesiotherapy and photomagnetic therapy [34]. It is worth noting that the studies included a course of treatment consisting of 10 procedures [24,32,34].

Given that photomagnetic therapy is a combined modality, the effects of this intervention are presented in both the magnetotherapy and laser therapy sections. In addition, the study by Kostro et al. compared magnetotherapy and laser therapy within the same study. Accordingly, its results are reflected in both the magnetotherapy section (for patients who received magnetotherapy) and the laser therapy section (for patients who received laser therapy). Thus, the results of these two studies are included in both the magnetotherapy and laser therapy sections. This categorization does not affect the total number of studies identified, which remains 12.

### 3.7. Laser Therapy

Four studies reported the use of this technique. Two were RCTs, and two were clinical studies. The treatment course lasted at least 10 days. The clinical effects achieved with this method were also widely reported. Given the different protocols for this physical agent, including treatment duration, treatment points, and wavelength spectrum, this method is widely used in patients with RA [24,27,33,34].

### 3.8. Cryotherapy

This review identified two cryotherapy techniques: local (5 min duration) and general (3 min duration). One study compared ice massage of the hands with cold-air treatment applied to the back and palm of the hand. Following this intervention, patients reported a reduction in pain [26]. A study by Klemm P et al. used general cryotherapy, which resulted in improvements in quality of life and reductions in disease activity and pain [31].

### 3.9. Psammotherapy

This procedure involves sand-bath treatment. This study examined the effect of this procedure on pain perception and manifestations, as well as kinesiophobia, over 3- and 5-day treatment periods. The study found that the impact of this factor on pain perception and intensity was significant in the 5-day treatment group [29].

### 3.10. Electrical Stimulation

Studies have examined the effect of electrical therapy on key indicators of rheumatoid arthritis disease activity. Despite differences in methodology, this type of treatment demonstrated positive effects in terms of pain reduction, decreased disease activity, and improved quality of life [23,25].

### 3.11. Wave Methods of Physiotherapy

Wave techniques are presented in two RCTs. The studies assessed their impact on pain severity and improvement in quality of life. Overall, the results of these studies demonstrate a beneficial effect on patients with RA [28,30].

Table 2 presents the main characteristics of the scale-based outcomes, key indicators, and limitations of the reviewed studies.

The results indicate that research in this field is ongoing among patients with RA. In addition to monotherapy approaches, combined treatment methods are also presented, which have also been shown to produce positive effects.

## 4. Discussion

The results of this literature review indicate the need for rehabilitation interventions for patients with rheumatoid arthritis at various stages of disease activity. The need to study rehabilitation support is particularly relevant for patients in the early stages of rheumatoid arthritis, which highlights the importance of timely diagnosis and comprehensive patient support.

Several authors also report an association between depression and rheumatoid arthritis, highlighting the need for psychological assessment in these patients [4,35]. Sleep disturbances can also be a manifestation of depression and anxiety.

This review showed that physiotherapy interventions are currently relatively uncommon and, in most cases, result in short-term positive effects. The lack of long-term follow-up after an intervention, despite existing knowledge of the mechanisms of action of specific physical factors, has important practical and clinical implications. Subsequent monitoring may reveal a peak therapeutic effect 2–4 weeks after a course of treatment with some physical factors. Therefore, issues related to the individual approach, the choice of physiotherapeutic interventions, their effectiveness, and long-term results are directly related to laboratory and clinical parameters assessed at the time of selecting the physiotherapeutic intervention, as well as to its technical characteristics.

It should also be noted that the literature widely describes various exercise therapy methods in review articles, systematic reviews, meta-analyses, and clinical studies, including clinical trials and RCTs [36,37,38,39,40,41,42,43]. This indicates an increased need to analyse existing exercise therapy programs for patients with RA and the potential for using various kinesiotherapy techniques. It should be noted that studies focusing exclusively on kinesiotherapy procedures were not included in this review.

However, compared with exercise therapy, alternative approaches involving physical factors in patients with RA are limited, which was one of the factors that motivated the development of this review.

### 4.1. Magnetic Therapy

One method of physical intervention is magnetic therapy. The primary mechanism of action is a magnetic field, which may be static or alternating, high-frequency or low-frequency. The clinical effect is thought to be associated with trophic, anti-oedema and anti-inflammatory effects. The search results demonstrate a positive trend in the use of magnetotherapy with regard to the indicators studied, such as a reduction in pain, an improvement in quality of life according to the HAQ index, and the ability to influence the duration of morning stiffness [24,32,34]. It should be noted that a static magnetic field may be more effective at reducing the duration of morning stiffness, whilst a pulsed field may help to reduce the severity of oedema and increase the mobility of the joint to which the physical stimulus was directed [24,32]. However, a 2016 review examining this factor in patients with RA noted an inconclusive effect, given the varying parameters of the procedures studied [19].

### 4.2. Laser Therapy

Laser therapy is widely used in patients with RA. The results of the identified studies demonstrate positive effects on key parameters of rheumatoid arthritis, including a reduction in the duration of morning stiffness, a decrease in pain intensity, and improvement in joint function. However, the literature contains mixed findings. For example, a systematic review of the effectiveness of laser therapy (including red and infrared lasers) found that the effect of infrared laser therapy may be comparable to that of placebo, whereas the effect of red laser therapy remains unclear [18].

On the other hand, a 2024 systematic review and meta-analysis reported a positive effect of low-level laser therapy on reducing the duration of morning stiffness and improving grip strength [44]. These differences may be due to variations in the methods and parameters used for this physical factor, which undoubtedly warrants further research and standardization of treatment protocols involving this physical factor. Evidence supporting the beneficial effects of experimental low-level laser therapy was also presented in a review by Hossein-Khannazer N. et al., which is consistent with the findings of the studies identified in our review regarding pain reduction [24,27,33,45].

The analysis of the identified studies revealed positive effects on pain severity and quality of life; the wavelength range was 632.8–830 nm. It is worth emphasizing that laser therapy can be combined with other methods, such as kinesiotherapy and magnetic therapy, as also demonstrated by the findings of our study [33,34]. This procedure can potentially be used in patients with RA; however, the heterogeneity of this technique requires further research involving larger samples and clearly defined patient selection criteria, exposure times, and treatment protocols, taking into account standardized data.

### 4.3. Electrical Stimulation

In our study, this category of intervention is represented by neuromuscular stimulation and neuroimmune modulation. It is worth noting that, despite the similarity of the physical factors, these methods differ significantly. For example, in a study by Piva et al., aerobic exercise was compared with passive stimulation of the quadriceps femoris, with both groups reporting positive results in terms of improved muscle structure and function [23]. Another study was an invasive trial in which vagus nerve stimulation produced positive results [25]. These studies also demonstrated a reduction in disease activity, as reflected by a decrease in the DAS-28 score, improved quality of life based on questionnaire assessments, and reduced pain [23,25].

We would also like to highlight other studies that were not included in this review but may be of interest when considering inflammatory diseases of the joint apparatus, such as osteoarthritis, given the presence of similar mechanisms underlying pain development and the possibility that rheumatoid arthritis may initially manifest in the knee joint.

For example, two RCTs have demonstrated the positive effect of this technique in patients with knee osteoarthritis [46,47]. The study by Mete O et al. proposes a technique of neuromuscular stimulation with voluntary movements (frequency 50 Hz, pulse duration: 300 microseconds (μs), duration 20 min), which was found to increase quadriceps strength and WOMAC scores compared with other methods [47]. Combination treatment in conjunction with physiotherapy also showed benefit in terms of improving WOMAC scores, which may indicate longer-lasting effects and is consistent with the studies presented in our work [23,25,47]. However, in the study by De Almeida CC et al., no advantage was demonstrated between the transcutaneous stimulation/interferential current/Australian current procedures and the placebo group; all groups noted an improvement in the WOMAC index [48]. Thus, this type of physiotherapy can also be presented in a combined method and can influence the improvement of patients’ quality of life and the functional state of the muscular system; however, the contradictions existing in the literature require further study of this factor.

### 4.4. Thermotherapeutic Methods

These thermal modalities include cold and heat treatments that affect microcirculation, nutrition, and pain. For example, cryotherapy, which has a short-term vasoconstrictive effect on the skin, has an anti-edematous and antispasmodic effect. Meanwhile, heat treatments ensure uniform tissue heating, promote vasodilation, improve tissue microcirculation and nutrition, and accelerate metabolic processes. These methods are actively used in patients with musculoskeletal disorders, as evidenced by a review study [20]. However, patients with RA require a rigorous, thorough examination to assess the disease’s phase, activity, and objective status. Contraindications for these procedures must be carefully considered and used with caution at various stages of the disease. Of importance is the potential neurological deficit in patients with RA, which can manifest as polyneuropathy and impaired temperature and pain sensitivity [49].

The cryotherapy procedure described in this study is limited to a number of sessions (n = 1), which precludes the assessment of subsequent effects, laboratory parameters, and functional status [26]. It should be noted that despite a single procedure, patients reported a reduction in pain. However, taking into account the data of another study presented as an RCT (n = 64), a positive analgesic effect was also noted [31]. General cryotherapy demonstrated a course of treatment and improvement in the following parameters: reduction in pain and disease activity, as well as improvement in quality of life. This is consistent with data from other studies. For example, a study by Guillot et al. examined the anti-inflammatory effect of cryotherapy on knee joints, some of which were patients with RA. A decrease in NF-kB p65 levels was noted, which may reflect disease activity and reduce pain [50].

Our search yielded a study on the use of psammotherapy, a traditional method involving sand-bath treatment. Following a strict methodology for performing this procedure, the best effects were demonstrated in the group that underwent a 5-day course of treatment, particularly in terms of pain reduction [29]. However, given the duration of the procedure, which can be up to 30 min, it is necessary to assess the condition of the cardiovascular and respiratory systems and the integrity of the skin before performing this procedure.

Heat therapy methods for RA are also extensively described in the review by Yao Y, including infrared therapy, therapeutic lamps, mud therapy, thermal water treatment, and combined heat therapy. The treatment courses described in the review lasted up to 4 weeks or for the duration of the arthritis. Twelve papers were analyzed in this study regarding the use of heat therapy in RA. A positive effect on quality of life was also noted, including a possible reduction in pain and improvement in functional activity [20]. This is consistent with the results obtained in the study by [29]. However, when performing cryotherapy and heat therapy procedures, it is necessary to follow safety regulations and carefully monitor the patient’s condition throughout the procedure.

### 4.5. Wave Methods of Physiotherapy

Wave-based treatments for RA include shockwave therapy and ultrasound therapy. The application of waves of varying frequencies to body tissues may improve microcirculation and metabolic processes, thereby affecting pain intensity. It is important to note the various physical parameters involved, which may result in differences in the number and duration of treatment sessions, penetration depth, and wave type and frequency.

This review includes RCTs demonstrating the effectiveness of ESWT. This technique demonstrated an analgesic effect, which is important for patients with RA. This was supported by laboratory findings demonstrating a significant reduction in inflammatory markers compared with another group [28]. This is consistent with the existing literature on research data in patients with RA.

A 2017 study presented the results of radial extracorporeal shockwave therapy for the reduction in arthralgia in patients with RA. Eleven patients discontinued their pain medication, while four reduced their dosage, which is consistent with the results of our search regarding shockwave therapy [51].

However, in the study by Imamura M. et al., it was found to be ineffective for patients with severe pain in patients with knee osteoarthritis (energy flux density: 0.10–0.16 mJ/mm^2^, 2000 pulses per session) [52].

The positive effect of extracorporeal shockwave therapy on the joint apparatus in patients with rheumatic diseases is supported by data from Phillip H. Yun and co-authors, taking into account the combination of pharmacological therapy and treatment during the period outside of disease exacerbation [53]. It is also worth noting that the frequency of shockwave therapy sessions differs from that of other physiotherapeutic methods, taking into account the specifics of this technique and the need for an interval between treatment sessions.

Ultrasound therapy. The RCT data presented in this review demonstrate improvements in ankle joint function, as well as muscle function. The authors also note that custom-made insoles can improve foot biomechanics, which is particularly important for maintaining a proper gait pattern [30]. However, the available literature is contradictory.

Research investigating the therapeutic effects of ultrasound on the musculoskeletal system remains limited. For example, a 2024 systematic review found conflicting results regarding the therapeutic effect of ultrasound therapy on shoulder pain and knee osteoarthritis. Five of seven studies reported improvements in the course and manifestations of osteoarthritis, but the results for the shoulder joint remained inconclusive [54]. A 2017 study also noted a reduction in pain and improvement in wrist joint mobility in patients with RA at an intensity of 0.7 W/cm^2^ for 7 min over a course of 10 sessions [55]. The efficacy data are consistent with the findings of the present study; however, it is necessary to clearly distinguish between different nosologies, because the treatment protocol depends on the specific clinical picture, including disease manifestations and activity.

It is important to note that the use of various rehabilitation methods allows patients to maintain regular contact with healthcare professionals, while also providing psychological and functional support. Patients are often left to cope with their illness on their own, seeking answers from unreliable online sources and thereby developing misconceptions and unnecessary concerns about their condition. Regular interaction with healthcare professionals enables patients to receive reliable information about disease progression and its characteristics, facilitating the selection of individualized non-pharmacological treatment strategies.

Thus, the identified studies describe a variety of methodological procedures that can be applied to patients with RA. Given the diversity of interventions and differences within single-method approaches, there is mixed evidence regarding the effectiveness of these factors, requiring further research. It should be noted that combination approaches can also be applied to patients with RA and represent promising future research.

### 4.6. Future Prospects

Given the wide variety of physical therapy methods based on physical factors, as well as the diversity of treatment methods, parameters, frequency, and duration, this area is of practical interest to healthcare. Further research requires a unified approach to standardizing intervention protocols, taking into account the stage and activity of RA, and increasing the number of sample sizes. Mixed intervention methods are also of great interest for exploring approaches to maintaining function and improving quality of life in patients with RA. It is important to bear in mind that the methods described in this paper have contraindications and side effects, which must be taken into account when prescribing treatments. Combined techniques also require careful consideration, as each patient’s level of physical fitness varies and the intensity of the treatment must be adjusted accordingly.

## 5. Conclusions

Physiotherapy appears to have a beneficial effect on the course of the disease; however, the available data do not allow a definitive assessment of its effectiveness due to the lack of long-term results and significant variation in the number of treatment sessions. Ongoing research indicates continued interest in this type of supportive treatment and its availability. However, small sample sizes in the included studies and heterogeneity among study groups with regard to disease activity and duration remain key determining factors. The long-term effects of physiotherapy interventions remain poorly understood and require the development of clear treatment protocols. Further research into these parameters is needed to identify the potential effects of therapeutic procedures in patients with rheumatoid arthritis.

Study Limitations. The limitations of this review include a small number of treatment sessions in some studies (1–2 sessions); heterogeneity of the physical factors used; differences related to the stage and activity of the disease; lack of long-term follow-up; differences in the number of treatment sessions; and the use of combined methods, which makes it difficult to determine the effectiveness of a specific physical factor. This review included both randomized and non-randomized studies, which may affect the interpretation of treatment effectiveness.

## Figures and Tables

**Table 1:** Physical therapy in patients with rheumatoid arthritis.

Physical Factor/Sample	Parameters	Number of Sessions	Result	Author
Physical agent modalities				
Neuromuscular electrical stimulation (31 people) /aerobic exercise (28 people)	-pulse frequency 75 per second, duration 450 microseconds. Two electrodes were placed proximally above the lateral vastus muscle of the thigh and one distally above the medial vastus muscle of the thigh, duration of 45 min. -aerobic exercise (36 training sessions in total), duration 45 min (min)	36 sessions over 16 weeks	In both groups, the following was observed: Improvement of muscle structure and functional capacity; reduction in pain and disease activity by −0.4 points and −0.5 points, respectively	Piva SR et al. [23]
Magnetic therapy/ laser therapy (30 people, 15 per group)	Laser therapy: power 450 milliwatt (mW), current strength 300 joule (J), dose 4.7 joule/square centimeter (J/cm^2^), duration 11 min. for each hand (joints of the hands) Magnetic therapy: frequency from 5 to 20 hertz (Hz), intensity from 3 to 5 millitesla (mT), duration of procedure from 15 to 20 min., alternating sinusoidal bipolar with a rectangular bipolar. (joints of the hands)	10	Reduction in pain syndrome; Increased range of motion in the joints of the hand and hand function, improved quality of life. The magnetotherapy group showed a noticeable advantage. Improved mobility in the interphalangeal joints	Kostro AM et al. [24]
Neuroimmune modulation, 242 participant’s Group 1: stimulation treatment, followed by a switch to open-label stimulation (122 patients); Group 2: placebo treatment, followed by a switch to open-label stimulation (120 patients) basic anti-inflammatory therapy	Fixation of the implant to the left cervical vagus nerve frequency 10 Hz, duration 1 min. (average value of 1.8 mA) Group 2:0 mA	Daily 3 months, followed by a transition to open stimulation	improvement in condition, reduction in disease activity; reduction in swelling and pain in joints	Tesser JRP et al. [25]
Cryotherapy (30 people): Group 1 (15 people) treatment with cold air at a temperature of minus 30° Celsius (C), Group 2 (15 people) ice massage of the hands	−30 °C (cold air) on the back and palm of the hand, at a distance of 15 cm; Group 2 received ice massage on the back and palm of each hand. Duration—5 min.	1	Pain relief	Laktašić Žerjavić N et al. [26]
Laser therapy (34 patients) Group 1: Low-intensity laser therapy (12 people), Group 2: Placebo laser + naproxen (12 people), Group 3: Naproxen 1000 mg per day (10 people)	Irradiation of 4 points: 1 laser wavelength 830 nanometers (nm), power 300 mW (irradiation of 3 points), 2 laser wavelengths: 632.8 nm, output power 7.3 mW (irradiation of 4th points) Points: pain point, acupuncture point on the hands, referred pain point, the C5–C7 region on the affected side for 15 min. each; for 4th point—1 min.	20 sessions 10 sessions per course, 10-day break (3 sessions per week)	Reduction in the duration of morning stiffness, number of painful and swollen joints in the group1	Al-Saraj MJA et al. [27]
Extracorporeal Shock wave therapy (ESWT): (40 people) Group 1: diclofenac 25 mg, 3 times a day and methotrexate (15 mg*once a week Group 2: SWT basic anti-inflammatory therapy	0.15–0.25 millijoule per square millimeter (mJ/mm^2^) at the beginning, depending on individual tolerance) and low frequency (less than 4 Hz), then the frequency was increased to 8 Hz for muscles and 4 Hz for tendons, with an exposure time of 30 min. Pressure 11–82 MPa, energy density—0.03–0.4 mJ/mm^2^. Before that, ultrasonic gel was applied. On the trigger point	2 sessions with an interval of 7 days	Significant pain reduction, immune response activation in group 1; reduction in depression and anxiety	Zhang M et al. [28]
Psammotherapy (30 patients: group 1—15 people for 3 days, group 2—15 people for 5 days). A total of 24 patients were included in the final analysis, as 6 withdrew for various reasons (3 did not complete the treatment, and 3 did not undergo the final assessment).	Sand bath—duration up to 30 min.	3 days 5 days	Reduction in pain intensity, an increase in the pain threshold in painful joints. Group 2 showed a noticeable advantage	Mahmoud NF et al. [29]
Underwater ultrasound. Three groups were included: Group 1 (25), the control group; Group 2 (25), the underwater ultrasound group; and Group 3 (25), the underwater ultrasound + insole group. The initial sample consisted of 75 participants, of whom 11 were lost to follow-up and 9 were excluded. The final analysis included 55 participants.	20 sessions of pulsed underwater ultrasound therapy. water temperature: 35–36 °C, Pulse mode, 0.9 W/cm^2^, Treatment area: ankle joint, 1 metatarsophalangeal joint, medial arch of the foot, time—7 min. (5 times a week). 3rd group was also provided with an insole	6 weeks	improvement in functional status	Özçelep Ö.F et al. [30]
Full body cryotherapy the main group (cryotherapy); the control group, total 64, final analysis: 56 people (1 participant dropped out due to infection, 7 participants without explanation)	cryosauna (1 session, temperature −130 °C, 50 Hz, duration: 1 session 90 s, 2–120 s, starting from the third session—3 min).	6 applications in 14 days	reduction in pain and decrease in disease activity	Klemm P et al. [31]
Combined methods				
Magnetic field + kinesiotherapy, Magnetic field therapy, total of 39 participants: 18 received treatment with a static magnetic field, and 21 received low-frequency pulsed electromagnetic therapy 32 people were included in the final analysis	Kinesiotherapy for all patients, 30 min. Static magnetic field (18), flux density 7 mT Low-frequency pulsed magnetic field (21), frequency 10–20 Hz, flux density 7 mT, using a rectangular bipolar pulse. Upper limbs (joints of the hand), Duration 20 min.	10	reduction in pain intensity by 2.2 points Static magnetic field—reduction in the duration of morning stiffness Low-frequency pulsed magnetic field— improvement in functional status and reduction in edema	Zwolińska J et al. [32]
Laser acupuncture (60 people): Group 1 (30 people) received remote-controlled laser acupuncture, aerobic exercise (tele-rehabilitation) + methotrexate; Group 2 (30 people) received aerobic exercise (tele-rehabilitation) + methotrexate;	808 nm, continuous wave, power 100 mW/cm^2^, energy density 7.5 J/cm^2^, irradiation period 60 s. irradiation of 4 acupuncture points + aerobic exercises, duration 20 min. 3 times a week	Course duration: 4 weeks, 6 sessions per week	improvement in quality of life in group 1; reduction in inflammatory markers	Adly AS et al. [33]
Photomagnetic therapy (72 patients): 43 people— photomagnetic therapy, 29 people (comparison group) against a background of standard treatment + and kinesiotherapy	Induction 12.5–25 mT, power density 3.5+/−0.5, wavelength 920–960 nm for infrared radiation, 610–680 nm for red radiation, localization—to the area of affected joints and blood vessels (inguinal, cubital), exposure time up to 30 min. (1 field— up to 10 min.)	Up to 10	Reduction in pain syndrome and duration of morning stiffness, increase in hand grip strength in the photomagnetic therapy group	Voichenko NV et al. [34]

C—Celsius; Hz—hertz; J—joule; J/cm^2^—joule/square centimeter; mA—Milliampere; mA/cm^2^—milliampere/square centimeter; mJ/mm^2^—millijoule per square millimeter; m—metre; MPa—megapascal; mT—millitesla; mW—milliwatt; mW/cm^2^—milliwatt/square centimeter; min—minutes; Nm—nanometer.

**Table 2:** Characteristics of the Studies.

Research	Type of Study	Sample Size	VAS/NRS/ Measurement Using an Algometer	HAQ/DASH /HAQ-20/HAQ-DI	DAS-28/DAS-28 CRP	Key Limitation	Effects
[23]	Randomized Pilot Study	59	*	↓	↓	long-term effect? sample size	improvement in muscle structure and function, *p* ranging from <0.001 to 0.019
[24]	Clinical trial	30	↓	↓	No data available	no control group; Precise localisation? limitations of the methodology	pain reduction (laser therapy *p* = 0.0046; magnetotherapy (*p* = 0.0014); improvement in quality of life, disabilities of arm, shoulder and hand (DASH) for group laserotherapy (*p* = 0.00019) and for group 2 (*p* = 0.00015).
[25]	pivotal Randomized Study, double-blind, placebo-controlled	242	*	↓	↓	invasive intervention	American College of Rheumatology 20% response criteria (ACR20) improvement 35.2%—1 group, 24.2—2 group, *p* = 0.0209 in 3 months; Up to 50% after assessment at 6 months, up to 52.8% at 12 months with active stimulation
[26]	Non-randomized clinical trial	30	↓	No data available	No data available after treatment	small sample, just 1 treatment	Reduction in pain from cold air and ice massage: *p* = 0.001 and *p* = 0.001
[27]	Randomized clinical trial, Single-blind	34	↓	No data available	↓	small sample, long-term effect?	reduction in: DAS28 score, *p* = 0.02, VAS *p* = 0.01, morning stiffness *p* = 0.05 ACR20-positive in the 1 group
[28]	Randomized controlled Study	40	↓	No data available	No data available	long-term effect? small sample	reduction pain *p* < 0.05
[29]	current observational cross-sectional study	30	↓	No data available	No data available	long-term effect?	reduction in pain intensity in the most painful joint, *p* < 0.05; increased pain threshold in the 3rd metacarpal joint and wrist, *p* < 0.05), in group 2
[30]	Randomized controlled Study, single-blind	75	No data available	↓	No data available	duration of the effect?	statistically significant differences in indicators: HAQ and Static Plantar Pressure Distribution (SPPD) *p* < 0.05
[31]	Randomized controlled trial, single-center, single-blind	64	↓	↓	↓	The treatment groups were not blinded;	Reduction in pain in the main group *p* < 0.001, in the control group- *p* = 0.003 after treatment. Reduction in pain in the main group *p* = 0.002 after 12 weeks; reduction in DAS-28 *p* = 0.004 in 12 weeks
[32]	Randomised Study, double-blind trial	39	↓	↓	No data available	long-term effect?	improvement in HAQ-20 score (*p* = 0.0166), reduction in VAS pain (*p* = 0.0000), reduction in duration of morning stiffness (*p* = 0.0010)
[33]	Randomized controlled trial	60	?	↓	No data available	long-term effect?	reduction points HAQ *p* < 0.05 in group 1
[34]	clinical trial	72	↓	↓	No data available	long-term effect?	HAQ reduction points 0.7 points (0.5–0.8) (*p* < 0.001) a 57.3% reduction in pain; a 58% reduction in the duration of morning stiffness; a 63.2% increase in hand grip strength

↓—decrease in the indicator; ?—there is no data on the scale used; however, indirect data may indicate a decline in the indicator; *—the pain in the legs was rated from 0 to 10; ACR20—American College of Rheumatology 20% response criteria; Anti-CCP—Anti-cyclic citrullinated peptide; CRP—C-reactive protein; DAS-28—Disease Activity Score using 28; DAS-28 CRP—Disease Activity Score using 28-joint count and C-reactive protein; HAQ—Health Activity Questionnaire; HAQ-DI—Health Assessment Questionnaire-Disability Index; NRS—Numeric Rating Scale; SPPD—Static Plantar Pressure Distribution; VAS—Visual Analogue Scale.

## Data Availability

No new data were created or analyzed in this study. Data sharing is not applicable to this article.

## Abbreviations

ACR20	American College of Rheumatology 20% response criteria
Anti-CCP	Anti-cyclic citrullinated peptide
CRP	C-reactive protein
C	Celsius
COVID-19	coronavirus disease
DAS-28	Disease Activity Score using 28
DAS-28 CRP	Disease Activity Score using 28-joint count and C-reactive protein
ESR	erythrocyte sedimentation rate
Hz	hertz
J	joule
J/cm^2^	joule/square centimeter
HAQ	Health Activity Questionnaire
mA	Milliampere
mA/cm^2^	milliampere/square centimeter
mJ/mm^2^	millijoule per square millimeter
m	metre
MPa	megapascal
mT	millitesla
mW	milliwatt
mW/cm^2^	milliwatt/square centimeter
min	minutes
NRS	Numeric Rating Scale
Nm	nanometer
RA	rheumatoid arthritis
RCT	Randomised clinical trials
RF	rheumatoid factor
ESWT	Extracorporeal Shock wave therapy
SPPD	Static Plantar Pressure Distribution
VAS	Visual Analogue Scale
WOMAC	Western Ontario and McMaster University Osteoarthritis Index
